# Tending Wounded Belligerents under the Protection of the Convention of Geneva

**Published:** 1912-12-14

**Authors:** 


### Tending Wounded Belligerents under the Protection of the Convention of Geneva.

### THE SOCIETIES ESTABLISHED IN FRANCE.

The following is an abstract of the "Simple Note" comprising the new rules for the French Red Cross Societies which was published in France, November 1912 :—

These Societies are three in number :—(1) The Society of Help to Wounded Soldiers, founded in 1866; (2) The Association of French Ladies, founded in 1879; (3) The Union of Women of France, founded in 1881.

These three Societies are recognised as of public utility and attached to the services of the Army by Presidential decrees. Their members receive in active employment the armlet bearing the Red Cross on a white ground; all three are under the Minister of War; no one Society in particular calls itself the French Red Cross. A Central Committee of the French Red Cross, comprising members of each of these three Societies, was constituted in 1907; this Committee is represented at the International Conferences, and deals with general questions bearing on the working of the three Societies.

### Statutes of the Association.

The statutes, approved by the Council of State, indicate the twofold end of the Association—help to the soldiers and sailors in case of war; help to the civil community in that of public disasters. To attain this end the Association prepares, by a special instruction system, a *personnel* of women who are thus rendered capable of affording efficacious and disciplined assistance to the wounded and sick of the Armies on land and sea. Secondly, it prepares, with all the care that contemporary science indicates, a store of dressings and auxiliary hospital material; to this effect it has established workrooms in each of its committees. Thirdly, it amasses a reserve fund for emergencies. Departmental committees, self-governing, while conforming to the statutes and regulations, carry out the same work as the Central Committee.

Touching the working of the Association in times of war it has thus been regulated by the decree of October 6, 1892 : "The Association of the French Ladies is authorised to second, in times of war, the Service of Military Health. For the accomplishment of this mission, it is placed under the authority of the commandment and directors of the Service of Health. The intervention of the Society is limited to the service of the territory; it can create auxiliary hospitals in the localities designated by the Ministry of War or by the generals commanding the territory, and lend its co-operation to the lines of communication in all that concerns the auxiliary field hospitals of that service. The *personnel* of the Society is authorised to carry the armlet badge of the Convention of Geneva. The legal independence of the Association was confirmed by a letter of the Minister of War dated June 18, 1890."

### Why the Association of French Ladies was Founded.

In 1877, seven years after the hard lessons of the Franco-German War, not one Society of Ladies was yet organised in France, while in Germany more than 60,000 women had responded to the appeal of the Empress Augusta and were strongly organised. Some of the Ladies' Committees which were improvised in France as auxiliaries of the Society of Aid to the Wounded in 1870 were dissolved after the war; they had never admitted the necessity of preparing their members by training of a special nature given in a permanent manner in times of peace. It was then that Dr. Duchaussoy (professeur agrégé at the Faculté de Paris) founded, with the concurrence of the Society of Practical Medicine, the first school for ambulance nurses and sick nurses in France. Encouraged by the excellent results from this instruction, he founded two years later, in 1879, the Association of French Ladies. This Association was authorised in 1881, recognised of public utility in 1883, and attached to the Ministries of War and the Marine in 1886. It was the first Society of Ladies of the Red Cross founded in France, and it was also the first which had a "School of Instruction for Lady Ambulance Nurses," and the first which introduced in its statutes the aid to the victims of public calamities.

The Association pursues with the greatest activity and success the organisation of *personnel* and stores of hospital auxiliaries in the territory for which it would be held responsible in the event of war. It has created a remarkable type by establishing the Hospital of Instruction of the French Ladies. This hospital has been erected at Auteuil; it affords 12,000 consultations and 7,000 dressings or operations annually, and is the first of its kind in France. Furthermore, for the purpose of affording practical instruction to the *Dames*, the Association has established several practical Dispensaries Schools.

In 1892, wishing to give youths of from 15 to 20 years and men freed from military service the means of serving their country usefully in the event of war, it founded a special instruction system for them, training them as stretcher-bearers, male sick nurses, tent erectors, and hospital stores clerks.

